# A Questionnaire Study to Investigate Stress among Future Pharmacists by Gender and Year Group

**DOI:** 10.3390/pharmacy6030075

**Published:** 2018-07-25

**Authors:** Lezley-Anne Hanna, Melissa Wilson, Maurice Hall, Alan Hanna

**Affiliations:** 1 School of Pharmacy, Queen’s University Belfast, 97 Lisburn Road, Belfast BT9 7BL, UK; mwilson71@qub.ac.uk (M.W.); m.hall@qub.ac.uk (M.H.); 2 Queen’s Management School, Queen’s University Belfast, Riddel Hall, 185 Stranmillis Road, Belfast BT9 5EE, UK; a.hanna@qub.ac.uk

**Keywords:** gender, pharmacy students, perceived stress, questionnaire, stressors, stress-coping

## Abstract

Background: This work aimed to ascertain future pharmacists’ stressors and stress-coping practices. Methods: Queens’ University Belfast Year 2 and 4 pharmacy students were invited to participate in an ethically approved, pre-piloted questionnaire study. Section A was the 10-item Perceived Stress Scale, Sections B and C related to stressors and stress-coping mechanisms, and Section D gathered non-identifiable demographic information. Data analysis largely took the form of descriptive statistics. Results: A response rate of 94.2% (213/226) was obtained. The mean Perceived Stress Scale score was 19.94 [standard deviation (SD) 6.37], with females having a higher mean score than males (20.55 SD 5.67 versus 18.16 SD 7.42). Common general stressors were career choice, employment opportunities, and finance. Common degree-specific stressors were particular assessments (objective structured clinical examinations and one-off written examinations) and the amount of course material. Popular stress-coping practices included getting emotional support from friends and family and using self-distractions. Conclusion: Stress appears to be an issue among these future pharmacists, and potentially more so for females. While the main stressors are unsurprising, this UK data enables comparisons to be made and helps inform support mechanisms within the university.

## 1. Introduction

Research has revealed that students can experience a substantial amount of stress, which in turn can lead to negative academic outcomes or health problems. Sources of stress (stressors) include academic-related issues (such as examinations or the clinical environment), personal factors (such as relationships), and their economic situation (such as debt and student loans) [1,2,3,4,5,6,7,8,9,10,11,12,13,14,15,16,17]. While there has been work conducted in this area involving medical students [8,9,10,11,12,13], dental students [14,15,16,17], and nursing students [6,7], it has not been researched to the same extent with pharmacy students. Indeed, to the best of the authors’ knowledge, sparse research has been carried out in the United Kingdom (UK) relating to stress among pharmacy students [18] (more studies have been conducted among pharmacy students in the United States of America (USA), such as ([4,19]), one study has been conducted in Malaysia [20], and one study has been conducted in Ghana [5]).

This current research is important, as it provides some baseline data from a UK context and therefore adds to the existing body of literature. It is anticipated that the work will help determine whether Queen’s University Belfast (QUB) School of Pharmacy needs to put further actions or mechanisms in place for its students in relation to stress. This work complements research that was conducted in the School about pharmacy students’ attitudes towards mental health [21] and a mental health first aid scheme and personal tutor scheme that have been implemented. In addition, the findings of this study could be useful to inform future teaching provision of the subject area within the School, since these students are future healthcare professionals who need to be able to provide accurate information on stress and its management to patients. 

The aim of this study was to investigate stress among QUB Master of Pharmacy (MPharm) students. The objectives were to determine students’ perceived stress levels using the Perceived Stress Scale and to investigate causes of stress (stressors) among students and ways they managed or coped with stress. Finally, the research sought to ascertain whether gender and year in the degree program affected responses. Establishing information on gender in the context of stress was of particular interest, as two of the academic authors (Lezley-Anne Hanna and Maurice Hall) had previously determined mean academic grades of the female and male students on the degree course and found that females outperformed their male counterparts academically, particularly in final year (Year 4). [The Year 2 mean grade (for Year 2 studies) was 64.73% (standard deviation 9.51) for females and 62.03% (standard deviation 8.19) for males, and the Year 4 mean grade (for Year 4 studies) was 71.62% (standard deviation 5.52) for females versus 65.36% (standard deviation 8.61) for males].

The principle findings of this research were that stress appears to be an issue among these future pharmacists, potentially more so for females. Popular stress-coping mechanisms included getting emotional support from friends and family and using distractions such as television rather than seeking medical advice or using pharmacological interventions. While the main stressors are perhaps unsurprising (career choice, employment opportunities, finance, particular assessments, and volume of course content to be learnt), this UK data enables comparisons to be made and helps inform support mechanisms within the university. 

## 2. Materials and Methods

All QUB Year 2 (second year of the four-year degree program) and Year 4 (fourth and final year of the program) MPharm students were invited to participate in the study. Year 4 students were chosen to represent ‘senior’ students (i.e., students who had almost completed the MPharm degree program and were soon to graduate) and Year 2 students were chosen to represent ‘junior’ students. Year 1 students were deliberately not chosen, as they had not undertaken any significant MPharm assessments at the time of the study (i.e., limited components had been completed and no written examinations had been undertaken), and part of this work aimed to investigate MPharm (degree specific) stressors. It was anticipated that there would be differences in perceived stress, stressors, and stress-coping mechanisms between these two year groups. 

Data was collected via a questionnaire. The paper-based, self-completed questionnaire was developed with reference to previous published work in the area [1,2,3,4,5,6,7,8,9,10,11,12,13,14,15,16,17,18,19,20]. The questionnaire consisted of four sections. The first section related to perceived stress. Several instruments have been used to measure stress [22,23,24,25], and one such instrument, the Perceived Stress Scale, measures perceived stress and reactions to stressful situations via ten statements [25]. As it is one of the most widely used psychological instruments for measuring the perception of stress, it was selected for use in this study and made up Section A of the questionnaire. It was not adapted in any capacity so that the findings of this current study could be compared with other relevant studies that had employed the Perceived Stress Scale. Section B focused firstly on general factors that may cause stress (stressors) such as financial situation, relationships, and health, and secondly on degree-related specific-stressors. The responders were asked to consider options and record answers against a 5-point rating scale (4 equated to ‘a lot of stress’ to 0 being ‘no stress’). There was a free response aspect to record other stressors that were not specifically mentioned. Section C related to stress-coping mechanisms, and respondents were asked to select as many stress-coping options as they wished from a comprehensive list. Again, there was a space for respondents to record other stress-coping mechanisms, should the list not suffice. Section D gathered three pieces of non-identifiable demographic information on gender, country where the student received most of their education prior to enrolling on the degree program, and year on the degree program (age was not ascertained as part of the questionnaire study, as it could have uniquely identified one or two individuals). From the School’s student records, the mean age of the Year 2 cohort was 20.75 years, and the mean age of the Year 4 cohort was 22.67 years in February 2018. A cover sheet at the start of the questionnaire outlined the purpose of the research, gave a predicted time required to complete the questionnaire, provided assurance the participation was voluntary, and explained how the data (which was non-identifiable) would be used. The cover sheet also stated that there were no awards or repercussions for completing or not completing the questionnaire. To maximize response rates, the questionnaire was relatively short and the questions were largely in a closed-question type style. The questionnaire was piloted on ten postgraduate students at the School. As a result of this pilot, an estimated completion time was ascertained, but no amendments were made to the questionnaire. Distribution of the questionnaires took place during December 2017 and January 2018. One author (Melissa Wilson) went to scheduled Year 2 and Year 4 compulsory classes, briefly introduced herself and the study, and invited students to participate. Students were asked to place completed questionnaires into the provided receptacle.

In terms of data analysis, the responses from the completed questionnaires were coded and entered into Microsoft Excel^®^ 2016 (Microsoft Corporation, Redmond, WA, USA) in February 2018. Given the respondents were essentially the entire population (rather than a random sample of a population from which inferences would be drawn), the analysis mainly took the form of descriptive statistics rather than inferential statistics. Further details about the Perceived Stress Scale scoring and analysis are provided in Table 1. In addition to Microsoft Excel^®^, R version: 3.4.1 (R Foundation for Statistical Computing, Vienna, Austria) was also employed for the data analysis [26].

The study was conducted in accordance with the Declaration of Helsinki, and the protocol was approved by the QUB School of Pharmacy Ethics Committee (Reference number: 024PMY2017; Date: 29 November 2017).

## 3. Results

### 3.1. Demographic Information (Section D of the Questionnaire) and Response Rate

A response rate of 94.2% (213/226) was obtained in the study. Initially, 218 surveys were returned, but 5 had to be discarded as they did not contain any of the three pieces of demographic information.

- Question 1 (gender): 27.2% (58/213) were male, 71.4% (152/213) were female, and 1.4% (3/213) did not disclose their gender.
  Please note: There were fewer male respondents, but this is reflective of the gender split of the students enrolled on the MPharm degree.
- Question 2 (year): 46.5% (99/213) were Year 2 and 53.5% (114/213) were Year 4.
- Question 3 (education background): 73.2% (156/213) received the majority of their education in the UK or Ireland prior to enrolling on the degree program; 23.9% (51/213) reported other countries or regions of the world, i.e., Asia, Greece, and Cyprus; and 2.8% (6/213) did not disclose this information.

### 3.2. Perceived Stress Scale Scores (Section A of the Questionnaire)

Perceived Stress Scale scores are shown in Table 1, including mean, standard deviation (SD), and median (firstly, for the whole cohort of respondents, then males and females, and finally Year 2 and Year 4). As the data were normally distributed (determined by the Shapiro-Wilk test), the Student’s *t*-test was used to compare overall means scores by gender and Year, with significance set at *p* < 0.05 *a priori*. There was a significant difference found in the gender mean scores (*p* = 0.03) but not Year mean scores (*p* = 0.68). However, in terms of effect size, Cohen’s d was small (d = 0.387) for gender and negligible (d = −0.058) for the Year comparisons. 

### 3.3. Stressors (Section B of the Questionnaire)

The three most commonly selected general stressors for the respondents (determined by interpolated medians, since the data were not normally distributed and ordinal [27]) were choice of career (interpolated median 2.00), future employment opportunities (interpolated median 1.86), and the student’s financial situation (interpolated median 1.82). The three most commonly selected degree-specific stressors were objective structured clinical examinations (OSCEs) (interpolated median 3.71), the volume of material to learn in the allocated timeframe (interpolated median 3.65), and one-off written examinations (interpolated median 3.53). 

The findings for each stressor (by gender and year group) are outlined in Figure 1 and Figure 2. As can be seen in Figure 1, females had higher interpolated medians than males for most stressors. Additionally, from Figure 2, some stressors of note that affected Year 2 more than Year 4 were oral presentations (2l), role plays with staff (2m), and practical classes (2k). 

Other general stressors (provided via free-response by a few respondents) included part time jobs and not having enough time to socialize with friends and family or take part in extracurricular and voluntary activities. Other degree-specific stressors (provided via free-response by a few respondents) included the Year 4 Responding to Symptoms and over-the-counter medicines component, the Year 4 Research Project, and the Year 2 extemporaneous dispensing component.

### 3.4. Stress-Coping Mechanisms and Practices (Section C of the Questionnaire)

**Figure 3:**
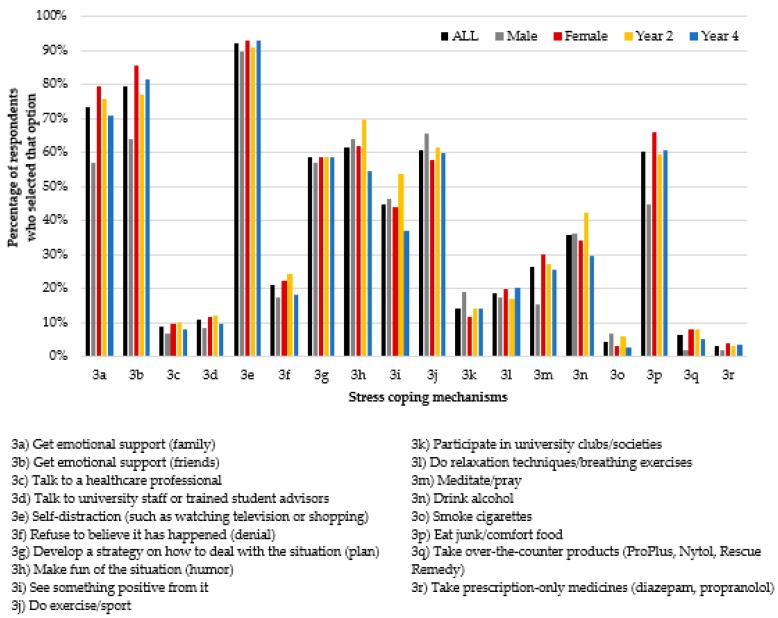
Stress coping mechanisms of student respondents (n = 213 for all; n = 58 male; n = 152 female; n = 99 Year 2 and n = 114 Year 4).

The findings relating to stress coping mechanisms are illustrated in Figure 3. As can be seen, the three most popular stress-coping mechanisms were self-distraction (3e) and getting emotional support from friends (3b) and from family (3a). More female students than males sought emotional support from friends (3b) and family (3a), comfort ate (3p), and meditated/prayed (3m).

## 4. Discussion

This work, relating to pharmacy students’ and stress, had several strengths and limitations. One of the main strengths was that this was one of few studies on stress to be performed on UK pharmacy students and to include comparisons by gender. Another strength was that a high response rate, meaning that non-response bias is unlikely. The study had limitations; for example, opinions on stress were only captured at one point in time, and scores and opinions may have differed had the study been performed closer to the end of year exams. Additionally, some statements were quite broad, such as the “choice of career” option (although this was not picked up at the pilot stage). Finally, the high female to male ratio is reflective of the student population in this one institution but could adversely affect the validity of the results. We invite readers to be aware of this and to contextualize the findings to their own educational or practice-based setting.

In this study, the mean Perceived Stress Scale score was 19.94, which is higher than that for members of the public [23] and consistent with students studying healthcare courses [28]. Female students had a higher mean score than males, which mirrors other studies performed on pharmacy students [5,24,29], medical students [30], and dental students [17,31]; however, it must be noted that our effect size was small.

The most common general stressor (determined by interpolated median) was the choice of career, closely followed by employment opportunities and the student’s financial situation. Choosing a career (and gaining employment) is undoubtedly a stressful process. However, the School of Pharmacy at QUB has a dedicated careers liaison academic who organizes careers fairs and provides students with information on UK pharmacy-related employment opportunities in hospital, community, industry, and academia. Moreover, job opportunities for pharmacy graduates in the UK are currently plentiful. Finance is a key stressor amongst university students throughout all disciplines [32], and this finding is consistent with studies performed on pharmacy students [29,33,34], medical students [35,36], and nursing students [37,38]. Being cognizant of, and managing, finances is a key life skill, so it is necessary to provide support to help nurture this.

Within the degree program itself, the most common stressors were OSCEs, one-off written examinations, and the large volume of material to learn within the allocated timeframe. OSCEs causing a high amount of stress and anxiety have been previously reported by Longyhore (2017) [39] and are consistent with previous work conducted in the QUB School of Pharmacy [40]. The volume of material to be learnt and clinical competency-type assessments are unlikely to change for quality assurance reasons; future pharmacists must be safe and effective practitioners. However, there may be scope to add in more formative assessments to help students gain confidence and experience in these in a safe environment. Finally, it is interesting to note that our students found group and individual assessments to be almost equally stressful; one might have hypothesized that individual assessments would be more stressful, as they tend to contribute to a larger part of the degree final grade than group work. Fostering effective team-working skills is imperative given the fact these future pharmacists will typically work as part of a wider healthcare team upon qualifying.

Female pharmacy students seemed to be more stressed by many of the degree elements compared with their male peers, which matched existing findings [19,41]. Other work has reported that males may consider revealing stress to be a sign of weakness [42], so this may partially help to explain the difference. Further research should be conducted to ascertain more about the role that gender plays with stress and its effect on academic performance. More Year 2 than Year 4 students considered degree aspects such as oral presentations, role play with staff, and practical laboratory classes stressful, perhaps because Year 4 students have more experience doing these aspects of the degree and hence may be better able to cope with them (i.e., find them less stressful). Peer support and mentoring (Year 4 students providing support to Year 2 students) could reduce some stress felt by these junior students, but any new intervention that is introduced should be investigated to measure impact and value.

The most popular stress-coping mechanisms and practices reported by the students were self-distraction and getting emotional support from both friends and family, which are similar to findings of a USA study involving pharmacy students [19]. In this current study, more female students than males sought emotional support from family and friends, which, again, could be because males are less likely to reveal that they are stressed in the first instance. The least popular stress-coping mechanisms were taking over-the-counter products, smoking cigarettes, and taking prescription-only medicines. These findings are potentially reassuring, i.e., these future pharmacists opted for social support and non-pharmacological measures such as exercise over pharmacological interventions to alleviate stress. Medicines and other substances used to alleviate stress will not necessarily resolve the underlying problem and can have side-effects including drowsiness and dependence.

## Figures and Tables

**Figure 1:** Stressors illustrated by gender (n = 152 females and n = 58 males, except 2k where n = 57).

**Figure 2:** Stressors illustrated by Year (n = 99 Year 2 and n = 114 Year 4, except 2k where n = 113).

**Table 1:** Perceived Stress Scale scores (per item and overall) for the respondents.

		A1 *	A2 *	A3 *	A4 *	A5 *	A6 *	A7 *	A8 *	A9 *	A10 *	Overall Score **
All	Mean	2.17	2.08	2.83	2.49	2.26	2.09	2.46	1.99	2.02	1.95	19.94
	SD	1.00	1.09	1.00	0.84	0.76	1.01	0.85	0.92	1.03	1.13	6.37
	Median	2	2	3	3	2	2	2	2	2	2	20
Male	Mean	1.88	1.91	2.60	2.69	2.29	1.88	2.57	2.02	1.74	1.71	18.16
	SD	1.06	1.23	1.14	0.90	0.84	1.14	0.88	1.00	1.02	1.20	7.42
	Median	2	2	2.5	3	2	2	3	2	2	2	19
Female	Mean	2.29	2.13	2.91	2.43	2.26	2.16	2.42	2.00	2.13	2.03	20.55
	SD	0.94	1.03	0.92	0.79	0.72	0.94	0.83	0.89	1.01	1.06	5.67
	Median	2	2	3	2	2	2	2	2	2	2	21
Year 2	Mean	2.10	2.10	2.85	2.40	2.17	2.21	2.49	1.94	2.00	1.89	20.14
	SD	1.04	1.05	1.05	0.84	0.81	1.08	0.88	0.92	1.09	1.11	6.60
	Median	2	2	3	2	2	2	3	2	2	2	20
Year 4	Mean	2.24	2.06	2.81	2.57	2.33	1.99	2.44	2.04	2.04	2.01	19.77
	SD	0.96	1.13	0.95	0.83	0.71	0.94	0.82	0.92	0.99	1.14	6.18
	Median	2	2	3	3	2	2	2	2	2	2	20

* Each item (A1–A10) is rated on a 5-point scale [never = 0 to almost always = 4]. ** Overall score is obtained by reversing the scores on the four positive items (i.e., A4, A5, A7, and A8) and then summing all 10 items.
